# Chemoattraction of macrophages by secretory molecules derived from cells expressing the signal peptide of eosinophil cationic protein

**DOI:** 10.1186/1752-0509-6-105

**Published:** 2012-08-20

**Authors:** Yu-Shu Liu, Pei-Wen Tsai, Yong Wang, Tan-chi Fan, Chia-Hung Hsieh, Margaret Dah-Tsyr Chang, Tun-Wen Pai, Chien-Fu Huang, Chung-Yu Lan, Hao-Teng Chang

**Affiliations:** 1 Graduate Institute of Molecular Systems Biomedicine, College of Medicine, China Medical University, Taichung, Taiwan; 2 Graduate Institute of Basic Medical Science, College of Medicine, China Medical University, Taichung, Taiwan; 3 China Medical University Hospital, Taichung, Taiwan; 4 Institute of Molecular and Cellular Biology, National Tsing Hua University, Hsinchu, Taiwan; 5 Academy of Mathematics and Systems Science, Chinese Academy of Sciences, Beijing, China; 6 Genomic Research Center, Academia Sinica, Taipei, Taiwan; 7 Department of Medical Science, National Tsing Hua University, Hsinchu, Taiwan; 8 Department of Computer Science and Engineering, National Taiwan Ocean University, Keelung, Taiwan; 9 Department of Biological Science and Technology, I-Shou University, Kaohsiung, Taiwan

**Keywords:** Eosinophil cationic protein (ECP), Signal peptide, Inflammation, Functional linkage network, Cell migration

## Abstract

**Background:**

Eosinophil cationic protein is a clinical asthma biomarker that would be released into blood, especially gathered in bronchia. The signal peptide of eosinophil cationic protein (ECPsp) plays an important role in translocating ECP to the extracellular space. We previously reported that ECPsp inhibits microbial growth and regulates the expression of mammalian genes encoding tumor growth factor-α (TGF-α) and epidermal growth factor receptor (EGFR).

**Results:**

In the present study, we first generated a DNA microarray dataset, which showed that ECPsp upregulated proinflammatory molecules, including chemokines, interferon-induced molecules, and Toll-like receptors. The levels of mRNAs encoding CCL5, CXCL10, CXCL11, CXCL16, STAT1, and STAT2 were increased in the presence of ECPsp by 2.07-, 4.21-, 7.52-, 2.6-, 3.58-, and 1.67-fold, respectively. We then constructed a functional linkage network by integrating the microarray dataset with the pathway database of Kyoto Encyclopedia of Genes and Genomes (KEGG). Follow-up analysis revealed that STAT1 and STAT2, important transcriptional factors that regulate cytokine expression and release, served as hubs to connect the pathways of cytokine stimulation (TGF-α and EGFR pathways) and inflammatory responses. Furthermore, integrating TGF-α and EGFR with the functional linkage network indicated that STAT1 and STAT2 served as hubs that connect two functional clusters, including (1) cell proliferation and survival, and (2) inflammation. Finally, we found that conditioned medium in which cells that express ECPsp had been cultured could chemoattract macrophages. Experimentally, we also demonstrated that the migration of macrophage could be inhibited by the individual treatment of siRNAs of STAT1 or STAT2. Therefore, we hypothesize that ECPsp may function as a regulator for enhancing the migration of macrophages through the upregualtion of the transcriptional factors STAT1 and STAT2.

**Conclusion:**

The increased expression and release of various cytokines triggered by ECPsp may attract macrophages to bronchia to purge damaged cells. Our approach, involving experimental and computational systems biology, predicts pathways and potential biological functions for further characterization of this novel function of ECPsp under inflammatory conditions.

## Background

Human eosinophil cationic protein (ECP) is an important molecule related to diseases such as asthma and inflammation. It is secreted by activated eosinophils and serves as one of the major components of eosinophil granule proteins [1]. Structurally, ECP contains three α-helices, five β-strands, and eight loops [2], with a molecular mass of 16–22 kDa, depending on the extent of post-translational modification [3,4]. Also called ribonuclease 3 (RNase3), ECP belongs to the human RNaseA superfamily, members of which have very low ribonucleolytic activity but high cytotoxic activity [5]. Members of the RNaseA superfamily are defined by their similar sequences and structures, and their ribonucleolytic activity regulates gene expression at the mRNA level [6]. To date, 13 human protein sequences have been found to be similar to bovine pancreatic RNaseA [7-10]. The 19 arginine and 2 lysine residues in the primary sequence of ECP account for its high isoelectric point (pI = 10.8) [11,12]. The highly positive charge carried by ECP contributes to its cytotoxic activity, as it facilitates interaction between ECP and negatively charged molecules, such as heparin sulfate and cell membrane lipids [11,13].

Through its interaction with carbonhydrate on cell surface, ECP translocates into cells by endocytosis and causes cell damage [13]. This may contribute to airway inflammation during asthma and to damage of the intestinal mucosa in Crohn’s disease [14-16]. The role of ECP in responding to pathogen infection by contributing to innate immune defenses through the removal of invading microorganisms is well documented. The protein is also cytotoxic to parasites, bacteria, viruses, helminths, and mammalian cells [15,17-19]. Therefore, ECP has been categorized as an antimicrobial peptide (AMP). The mechanism of ECP-triggered cell damage is suggested to involve destabilization of cell membrane lipids through pore formation, causing changes in membrane permeability and membrane leakage [5,12,20].

Human ECP possesses a 27-amino-acid signal peptide that is cleaved from the rest of the protein by a signal peptidase (SP) in the endoplasmic reticulum (ER). The signal peptide of ECP (ECPsp), which comprises a short, positively charged N-terminal region, a central hydrophobic region, and a polar C-terminal region, leads to the secretion of ECP into the extracellular space. The cytotoxicity of ECPsp inhibits the growth of lower organisms, such as *Escherichia coli* and *Pichia pastoris*, but not that of mammalian cells [21]. This growth inhibition results from the suppression of *de novo* protein synthesis. Cell growth is restored when signal peptide peptidase (SPP) and ECPsp are co-expressed in *P. pastoris*[21]. Cleavage of ECPsp into the ECPsp-1-17 and ECPsp-18-27 fragments by SPP eliminates the cytotoxicity, thus preventing damage to mammalian cells. Furthermore, knockdown of the SPP mRNA level in mammalian cells restored the inhibitory effect of ECPsp on cell proliferation [21].

Moreover, in 2007 we reported ECPsp-1-17 enhances the expression of tumor growth factor-alpha (TGF-α) and epidermal growth factor receptor (EGFR) at both the mRNA and protein levels in A431 and HL-60 cell lines [22]. Therefore, ECPsp may possess dual functions involving both protein secretion and the regulation of gene expression. In this study, we use a systems biology approach to further characterize the biological function(s) of ECPsp. In particular, we used DNA microarray analysis to investigate the ECPsp-triggered gene expression and to characterize the genome-wide interactome of ECPsp. By combining these two datasets, we explored the integrated network from STRING 9.0 and KEGG to identify potential pathways and functional clusters to profile ECPsp-induced gene expression. Finally, we used a cell migration assay to demonstrate the novel functions of ECPsp that were discovered using the systems biology approaches.

## Results

### Inflammatory molecules upregulated by ECPsp are central in the functional linkage network

**Figure 1:**
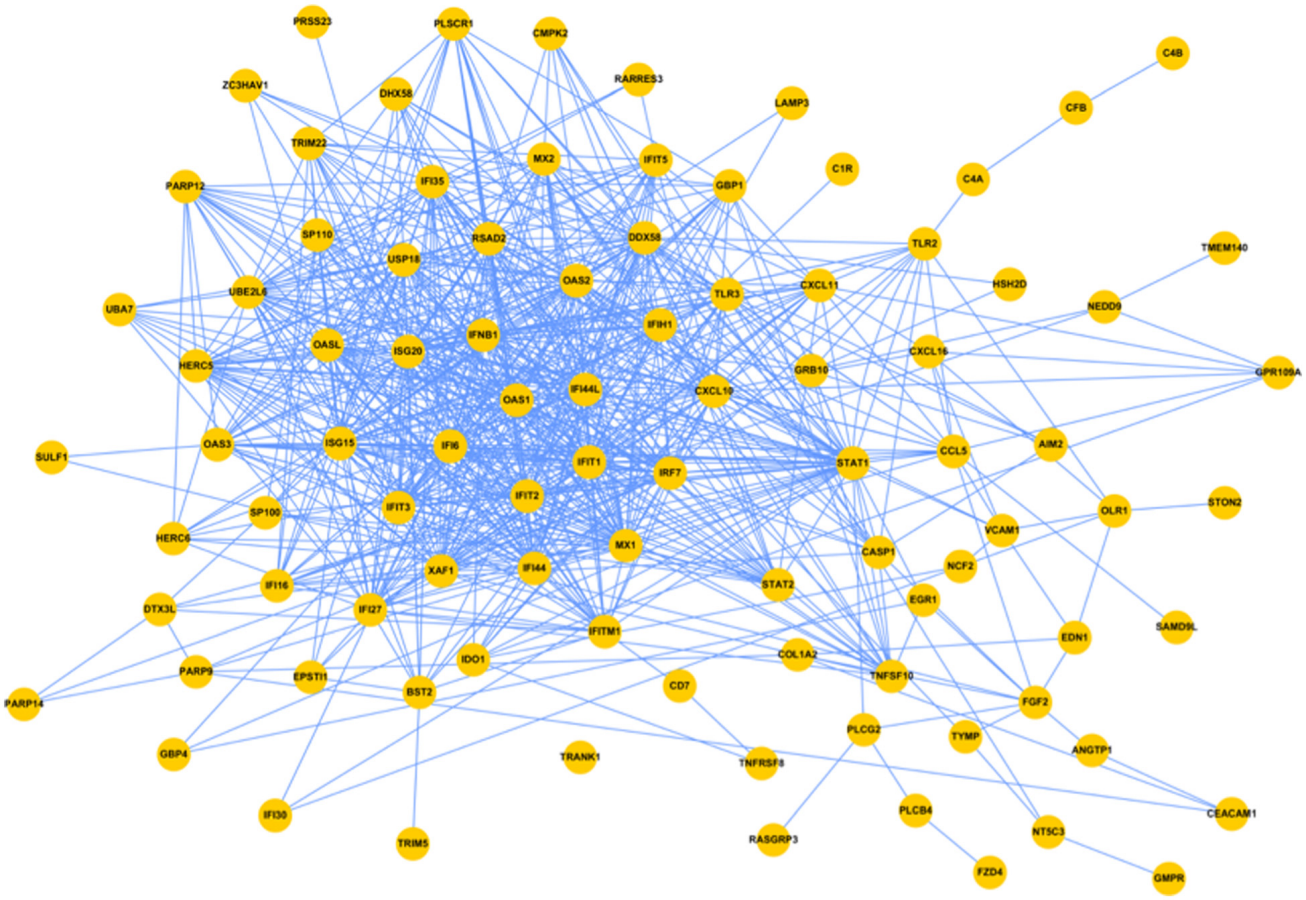
**Functional linkage network of ECPsp-induced genes.** The nodes in the network are the ECPsp-induced genes for which their expression in ECPsp-eGFP/BEAS-2B cells was at least twofold that in the control eGFP/BEAS-2B (also see Table 1). The edges in the network are modified from STRING.

To understand the gene expression profile triggered by ECPsp, we used DNA microarray technology to analyze the transcriptome. All microarray analyses were conducted by Phalanx Biotech (Taiwan). Each total RNA, derived from the cells transfected with control plasmids or plasmids containing ECPsp, was duplicated on the array. We performed two technical replicates of all experiments to confirm the data and account for experimental variations. According to the gene expression data, 93 genes were upregulated by more than twofold with a statistics *p* value less than 0.05 in ECPsp-expressing cells relative to controls (Table 1). The 93 genes were submitted to the functional linkage network obtained from the STRING 9.0 database, and a network was generated by choosing the default setting (Figure 1).

**Table 1:** Genes induced by ECPsp more than twofold relative to the control

**Gene Symbol**	**Fold**	***p-value***	**Gene symbol**	**Fold**	***p-value***
IFI44L	5.627	6.13E-04	FZD4	2.470	4.53E-06
CMPK2	5.477	5.24E-07	SULF1	2.455	8.71E-03
RSAD2	5.401	2.36E-06	TNFRSF8	2.452	4.53E-04
CXCL11	5.269	1.02E-04	IFIT5	2.439	3.46E-04
CCL5	4.823	6.01E-05	IFI30	2.431	3.64E-03
ANGPT1	4.542	1.11E-05	EGR1	2.423	6.42E-05
TNFSF10	4.531	2.63E-04	IFI35	2.413	1.26E-03
SAMD9L	4.208	6.56E-08	VCAM1	2.412	8.27E-04
IFIH1	4.199	3.38E-08	GBP1	2.397	4.11E-10
ISG20	4.169	1.14E-04	FGF2	2.385	2.62E-15
IDO1	3.956	1.50E-04	IFI16	2.360	4.28E-06
OAS1	3.919	2.71E-03	STAT1	2.350	2.85E-06
TLR3	3.908	2.72E-07	PLSCR1	2.331	4.79E-05
MX2	3.860	5.31E-04	IRF7	2.305	3.98E-03
CXCL10	3.827	6.56E-10	CD7	2.304	2.87E-04
HERC5	3.783	2.17E-07	IFI27	2.285	3.08E-02
RASGRP3	3.745	2.65E-05	DHX58	2.276	5.73E-04
OAS2	3.695	1.56E-04	PLCG2	2.251	6.55E-07
DDX58	3.596	5.24E-09	UBE2L6	2.248	8.97E-04
XAF1	3.522	1.65E-04	IFI6	2.243	3.62E-02
IFIT2	3.496	2.09E-09	BST2	2.222	2.91E-02
GPR109B	3.435	6.36E-05	NCF2	2.216	1.03E-13
LAMP3	3.295	3.38E-04	ZC3HAV1	2.210	4.61E-06
GBP4	3.194	1.68E-07	PLCB4	2.204	3.35E-10
OASL	3.074	1.79E-05	EDN1	2.204	4.94E-02
HERC6	3.027	8.91E-06	C1R	2.203	4.43E-02
TRIM22	2.961	6.67E-08	COL1A2	2.193	3.44E-03
USP18	2.952	8.08E-04	SP100	2.185	3.74E-08
SP110	2.941	1.82E-06	PARP12	2.185	3.27E-05
MX1	2.891	9.80E-04	GRB10	2.177	5.06E-10
IFI44	2.863	8.60E-08	TLR2	2.163	1.86E-03
PARP9	2.859	8.82E-03	ISG15	2.148	4.58E-04
IFIT1	2.843	1.36E-04	DTX3L	2.134	3.69E-06
IFIT3	2.819	1.91E-04	IFNB1	2.122	4.83E-05
OLR1	2.811	8.00E-05	UBA7	2.116	1.62E-02
TMEM140	2.802	2.26E-04	TYMP	2.105	2.76E-02
HSH2D	2.786	1.43E-02	NT5C3	2.097	2.00E-05
TRANK1	2.759	5.27E-04	GMPR	2.080	1.90E-04
PARP14	2.741	3.27E-07	OAS3	2.069	6.65E-04
STAT2	2.687	2.46E-05	AIM2	2.060	2.46E-07
CASP1	2.678	2.02E-03	CXCL16	2.046	9.54E-04
IFITM1	2.660	4.49E-03	PRSS23	2.028	6.38E-12
CEACAM1	2.612	4.38E-05	TRIM5	2.010	1.12E-03
NEDD9	2.529	1.75E-03	STON2	2.009	1.13E-02
RARRES3	2.520	4.00E-08	C4A	2.006	3.18E-02
EPSTI1	2.484	3.17E-03	C4B	2.006	3.18E-02
CFB	2.470	2.39E-02			

Within this sub-network, a clearly evident area of dense interconnections contains nodes that are related to inflammatory responses. These include chemokines, interferon-induced/related molecules, and inflammatory receptors, such as Toll-like receptors. The focused area thus reveals that ECPsp may induce the expression of genes that encode proinflammatory molecules for triggering inflammatory pathways. This discovery is crucial to understanding the biological function of ECPsp. The function of ECPsp as a conventional signal peptide for the secretion of mature ECP is well documented [23]. We used differential display technique and real-time RT-PCR to demonstrate that ECPsp induces TGF-α and EGFR overexpression [22]. Another study indicated that ECPsp is cytotoxic to bacteria and yeast, but not to mammalian cells [21]. These findings demonstrated that ECPsp regulates gene expression in addition to executing its conventional function, protein secretion.

Patients with asthma usually experience severe inflammation in the respiratory tract. The activated eosinophils that gather in the airways release mature ECP, which in turn damages bronchial epithelial cells. At the same time, it is possible that ECPsp might induce and release cytokines and chemokines to attract immune cells, such as T cells, NK cells, eosinophils and macrophages.

**Figure 2:**
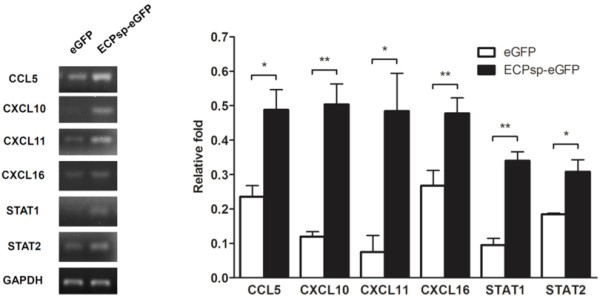
**Upregulation of proinflammatory molecules in BEAS-2B cells.** The levels of mRNAs that encode CCL5, CXCL10, CXCL11, CXCL16, STAT1, and STAT2 were monitored using semi-quantitative RT-PCR. A gel showing the results from one representative experiment is shown on the left. The relative level was determined by normalization against the level of mRNA encoding GAPDH, using IMageJ software. The average, with standard deviation, was derived from three independent experiments. * *p* <0.05; ** *p* <0.01.

To explain the ontology of the functional network, Network Ontology Analysis (NOA) which applied the categories of Gene Ontology to network analysis [24] would be employed. In the NOA results, the category ‘biological process’ is related to inflammation that is involved in the immune response, including viral infection and biotic stimulation. The ‘cellular component’ is consistent with the extracellular release of cytokines, chemokines, and interferons. Finally, the ‘molecular function’ is related to the production of chemokines and cytokines and activation of the JAK/STAT (signal transducers and activators of transcription) signaling pathway, which is involved in interferon signaling and chemokine production (Table 2) [25]. To confirm the upregulation of molecules related to chemokine and JAK/STAT signaling, the mRNA levels of CCL5, CXCL10, CXCL11, CXCL16, STAT1, and STAT2 were measured by semi-quantitative RT-PCR. Indeed, levels of mRNAs encoding CCL5, CXCL10, CXCL11, CXCL16, STAT1, and STAT2 were upregulated 2.07-, 4.21-, 7.52-, 2.6-, 3.58-, and 1.67-fold, respectively, in cells that expressed ECPsp-eGFP as compared with cells that expressed eGFP only (Figure 2). 

**Table 2:** The top four functional linkage networks identified by NOA

**Gene ontology**	**GO: Term**	***p*-value**	**Term name**
Biological process	GO:0009615	2.1E-24	response to virus
	GO:0009607	1.3E-20	response to biotic stimulus
	GO:0051707	9.7E-20	response to other organism
	GO:0002376	4.3E-19	immune system process
Cellular component	GO:0005615	1.8E-6	extracellular space
	GO:0005737	9.7E-6	cytoplasm
	GO:0044421	1.0E-4	extracellular region part
	GO:0005576	0.0023	extracellular region
Molecular function	GO:0005062	3.0E-6	hematopoietin/interferon-class (D200-domain) cytokine receptor signal transducer activity
	GO:0016763	4.9E-6	transferase activity, transferring pentosyl groups
	GO:0003950	1.1E-5	NAD^+^ ADP-ribosyltransferase activity
	GO:0008009	1.2E-4	chemokine activity

### Integrating insights into ECPsp-mediated gene induction with knowledge of TGF-α/EGFR signaling

We previously demonstrated that ECPsp induced TGF-α and EGFR expression at the mRNA and protein levels in A431 cells [22]. The growth factor TGF-α is one of the ligands that stimulates EGFR phosphorylation and triggers the signaling pathways downstream of this event. Since the analysis of the KEGG database revealed that the STAT pathway is triggered by TGF-α *via* EGFR, resulting in cell proliferation, cell survival, migration and invasion, and the STATs could be activated by chemokines and chemokine receptors and then drive the expression of cytokine-encoding genes, we hypothesized that STATs may serve as key transcriptional regulators in the inflammatory response triggered by ECPsp. Thus, incorporation of TGF-α, EGFR, and the ECPsp-induced genes may enable the discovery of new regulatory networks initiated by ECPsp. To reduce the complexity of this analysis, the area of dense interconnections in Figure 1 was extracted and combined with TGF-α and EGFR signaling pathway components.

**Figure 3:**
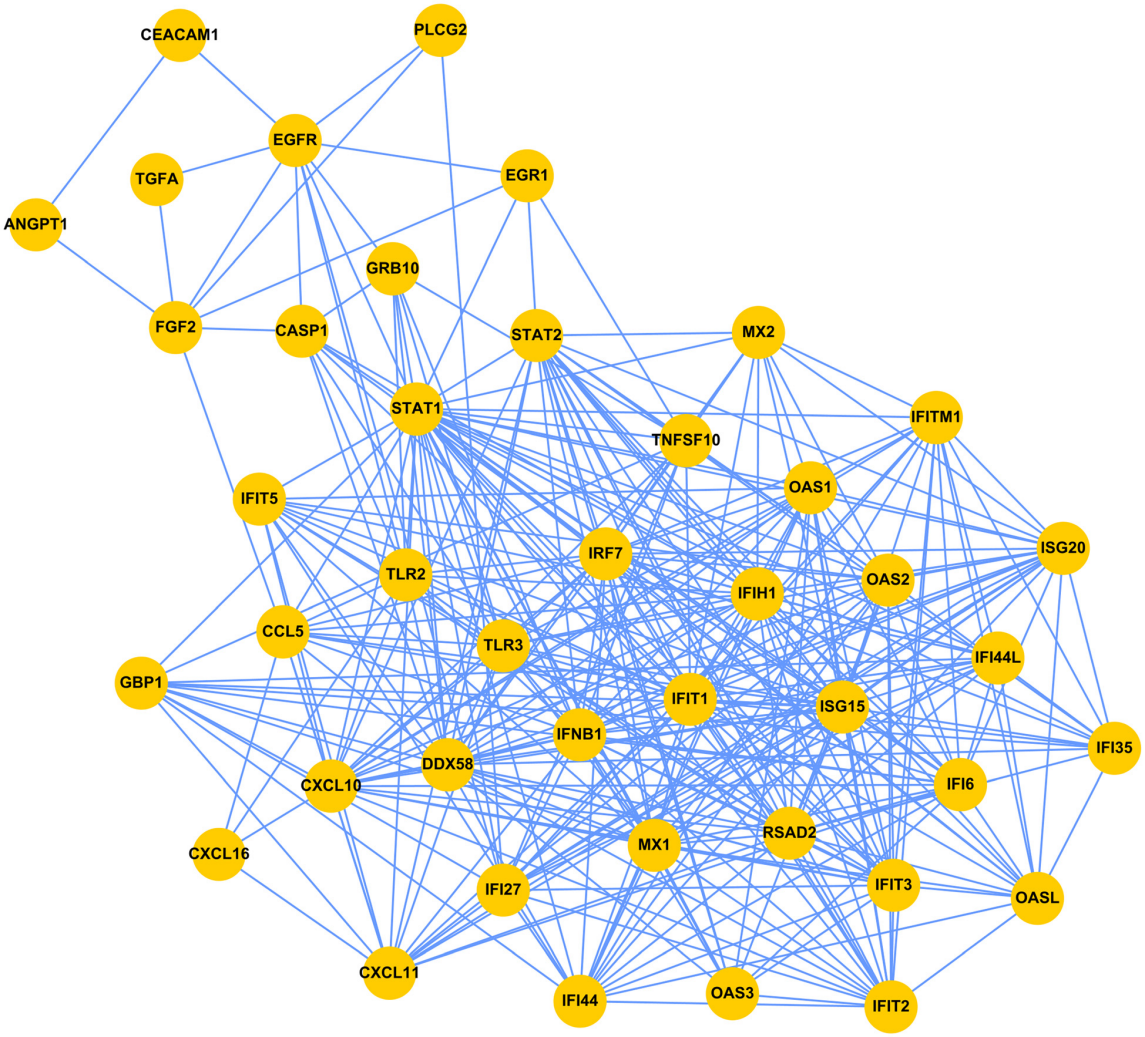
**Proposed functional network combining features of the focused ECPsp network with TGF-αand EGFR signaling components.** To connect the data obtained using gene expression profiling with additional experimental data, we selected molecules from the area of dense interconnections in Figure 1 and incorporated these with known features of TGF-α and EGFR signaling.

The area of dense interconnections was determined manually, which was enlarged and extracted from a seed of IFNB1 to the borders of a proinflammatory molecule and a non-proinflammatory molecule. There are 40 nodes in the area of dense interconnections. After their incorporation, the resulting network showed that the TGF-α/EGFR pathway connected with inflammatory molecules *via* STAT1 and STAT2, important transcriptional factors that regulate cytokine expression and release (Figure 3) [26]. Therefore, ECPsp may initiate alternative inflammatory responses in cells when ECP is expressed simultaneously at asthma/inflammatiory condition. In addition, within these 41 nodes, 9 (CEACAM1, PLCG2, EGR1, GRB10, CASP1, FGF2, ANGPT1, and TNFSF10) were not related with inflammatory responses. However within the 9 nodes, 7 (CEACAM1, PLCG2, EGR1, GRB10, CASP1, FGF2, ANGPT1) were located within the TGF-α/EGFR pathway network and these nodes functioned as cell proliferation, angiogenesis, and apoptosis, corresponding to the proposed functions of this subnetwork. The other two nodes, TNFSF10 and DDX58, were not proinflammatory molecule within the proinflammation network composed with 34 genes. Thus we concluded that within the proinflammation network, 94.12% (32/34) of nodes were inflammation-related ones. 

Since it is hypothesized that STAT1 and STAT2 might serve as key regulators between the subnetworks in BEAS-2B cells, we also investigated the mRNA and protein levels of STAT1 and STAT2 in A431 cells in which the TGF-α and EGFR are upregulated by ECPsp. If the STAT1 and STAT2 also upregulated by ECPsp, it is confident to merge the TGF-α/EGFR signaling network and the proinflammatory network. As shown in Additional file 1: Figure S1, the levels of mRNAs of STAT1 and STAT2 were increased in the presence of ECPsp by 1.67- and 1.57-fold, respectively, as well as the protein level by 1.54- and 2.72- fold, respectively. These results indicated that STATs might serve as real hubs to connect two subnetworks and regulate the gene expression induced by ECPsp.

### STAT1 and STAT2 serve as hubs that connects TGF-α pathways and cytokine/chemokine production

**Figure 4:**
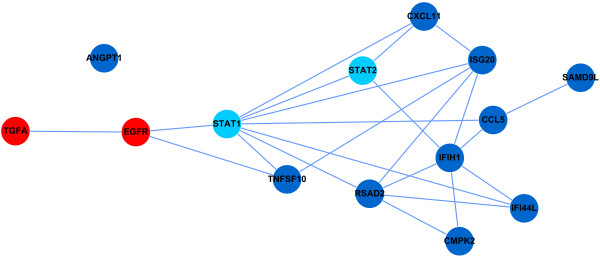
**STAT1 interconnects genes with high expression levels, TGF-αand EGFR.** Genes with a greater than fourfold increase in expression in the presence of ECPsp are indicated by dark blue circles, whereas STAT1/2 are indicated by light blue circles, and TGF-α and EGFR are indicated by red circles.

To obtain a high-confidence network, we further studied the molecules that were induced by ECPsp by more than fourfold. The underlying functional linkage network is shown in Figure 4. The functional linkage network was combined with the current knowledge of TGF-α, EGFR, and STAT1/2 signaling. As shown in Figure 4, STAT1 and STAT2 still serve as hubs that connect two functional clusters. One cluster contains interferon-inducing factors and chemokines, such as IFI44L, IFIH1, CCL5, and CXCL11. Another cluster comprises molecules related to cell growth and proliferation, such as TGF-α and EGFR. This network provides potentially valuable avenues for future research concerning the novel functions of ECPsp.

### Medium conditioned by ECPsp-expressing cells causes migration of RAW 264.7 cells

**Figure 5:**
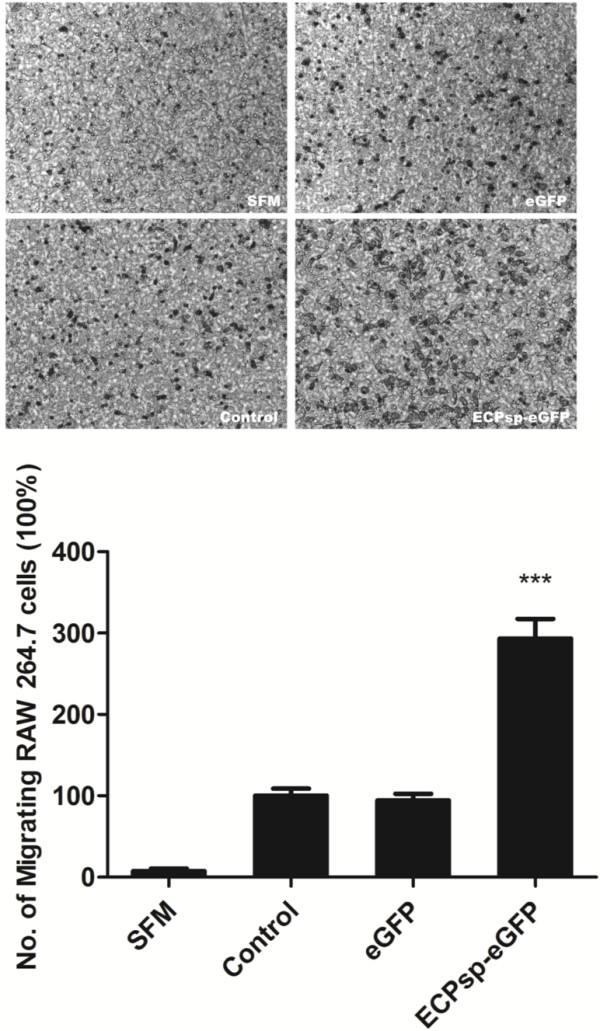
**Migration of RAW 264.7 cells is increased in ECPsp-conditioned medium.** The macrophages were chemoattracted by serum free medium (SFM, upper left), conditioned medium culturing BEAS-2B cells (Ctrl, lower left), conditioned medium culturing eGFP/BEAS-2B cells (eGFP, upper right) and conditioned medium culturing ECPsp-eGFP/BEAS-2B (ECPsp-eGFP, lower right). The folds of migrating RAW 264.7 cells is increased in ECPsp-conditioned medium by 2.93- and 3.11-fold relative to medium conditioned by BEAS-2B and eGFP/BEAS-2B cells, respectively. The average, with standard deviation, was derived from three independent experiments. *** *p* <0.001.

**Figure 6:**
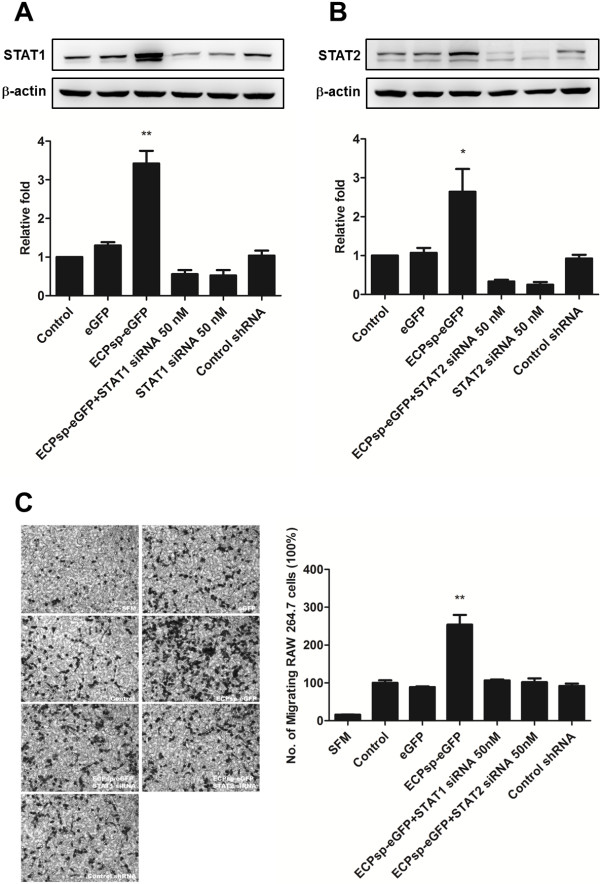
**Knockdown of STAT1 and STAT2 results in the decrease of migration of RAW 264.7 cells.** The protein expression of STAT1 (A) and STAT2 (B) was knocked down using individual siRNA. The fold of migrating RAW 264.7 cells is decreased in the medium conditioned by BEAS-2B cells cotransfected with ECPsp-eGFP and STAT1 or STAT2 siRNA by 2.38- and 2.50-fold relative to medium conditioned by BEAS-2B, respectively. The average, with standard deviation, was derived from three independent experiments. * *p* <0.05; ** *p* <0.01.

Our observation that the expression of genes encoding the chemokines CCL5, CXCL10, CXCL11, and CXCL16 is induced by ECPsp suggests that cells expressing ECPsp may induce chemoattraction. Among these four chemokines, some may be responsible for novel biological functions of ECPsp. Several reports show that CXCL10 [27], CXCL11 [28], and CXCL16 can initiate the homing of Th1 cells, NK cells, and eosinophils [29] and that CCL5/RANTES attracts macrophages and eosinophils [30-33]. We assayed macrophage migration using the human macrophage-like cell line RAW 264.7 to investigate whether the cytokines produced by ECPsp regulate the recruitment of immune cells. Compared with medium conditioned by culturing ECPsp/BEAS-2B and eGFP/BEAS-2B cells, the respective rates of chemoattractant-mediated macrophage migration were 2.93- and 3.11-fold higher, respectively, for medium conditioned by cells expressing ECPsp-eGFP (Figure 5). Given that ECPsp is expressed by activated eosinophils at sites of inflammation or infection, secretory chemokines might attract macrophages to eliminate damaged cells or pathogens. This is the first time that ECPsp has been proposed to function not only in secretion but also in assisting immune-cell migration. In order to demonstrate whether STAT1 and STAT2 serve as key transcriptional regulators or not experimentally, the individual siRNA against STAT1 or STAT2 was transfected into BEAS-2B cells which express eGFP or ECPsp-eGFP. Figure 6 reveals that while the mRNA and protein levels of STAT1 and STAT2 were knocked down, the migration of macrophages, which was induced by ECPsp, was downregulated by 2.38- and 2.50-fold, respectively. It indicated that the migration of macrophages enhanced by ECPsp was indeed regulated *via* STAT1 and STAT2. It might reflect our hypothesis that the STAT1 and STAT2 serve as hubs to regulate the gene expression of chemokines and cytokines in the ECPsp/BEAS-2B cells. 

## Discussion

In this study, we generated the functional network using STRING combined with KEGG. Additionally, we also analyzed the 93 genes using the network dataset generated by Cui and colleagues [34]. Cui’s dataset, including 1,634 genes, is comprehensive for analyzing signaling pathways in cancers. However, only 15 genes overlap with our 93 genes. The signaling network could not be generated using these 15 genes. It may be due to our dataset is related with proinflammatory effects and Cui’s dataset is focused on cancer signaling.

Under asthmatic conditions, ECP would be released by activated eosinophils and damage bronchial epithelial cells. At the same time, ECPsp is cleaved by human SP and SPP, and the resulting ECPsp triggers overexpression of TGF-α and EGFR. It suggested that the short signal peptide of ECP might function not only in protein secretion but also to regulate gene expression [22]. Similarly, the signal peptide may provide other functions besides protein targeting. Localization of the signal peptide of the envelope glycoprotein (Env) of Jaagsiekte sheep retrovirus to the nucleus regulates viral gene expression by mediating a beneficial post-translational step in the replication cycle [35]. In addition, interaction of calmodulin with the signal peptide fragments of preprolactin and HIV-1 gp160 processed by SPP triggers Ca^2+^/calmodulin-dependent cellular signaling [36].

Our study provides evidence that ECPsp does indeed regulate gene expression in cells and that the upregulated genes participate in inflammation-related processes. Under inflammatory conditions, eosinophils might release cytokines to stimulate cell growth and activate immune cells; they might release chemokines to attract macrophages to the bronchia to eliminate the pathogens and damaged cell debris after secretion of ECP. The present microarray profiles show the upregulation of four chemokines, namely CCL5, interferon-induced protein of 10 kDa (IP-10/CXCL10), interferon-inducible T-cell alpha chemoattractant (I-TAC/CXCL11), and CXCL16. Also called RANTES, the chemotactic cytokine CCL5 is the most potent of these four chemokines, as it is able to both recruit eosinophils to tissues (*via* binding to CCR3) and attract macrophages (*via* CCR5), Th1 cells, and basophils to the site of inflammation [30-33]. Moreover, CXCL10 binds CXCR3 and enrolls Th1 cells to resist intracellular pathogen infection, such as viruses [27]. In addition, CXCL10 is an important contributor to airway hyperactivity [37]. Another ligand of CXCR3, CXCL11, attracts Th1 cells, NK cells, and eosinophils to inflammatory sites [28]. The cytokine CXCL16 interacts with CXCR6 on Th1 and CD8 effector T cells and plays a crucial role in recruiting these cells to sites of inflammation [29]. Apart from the molecules mentioned above, we observed IFN-β and many interferon-related molecules in the dataset generated by DNA microarray analysis. Interferons have several functions in common, many of which involve anti-viral and anti-tumor activities. By interacting with their specific receptors, interferons can activate signaling pathways transmitted by STAT proteins. The STATs (STAT1–STAT6) are a family of transcriptional factors that regulate the expression of particular immunoregulatory genes. They can be activated by type I (IFN-α and IFN-β), type II (IFN-γ), and type III (IFN-λ) interferons [38,39]. For example, type-I IFNs can induce gene expression *via* either the ISRE (interferon-sensitive response element) or GAS (gamma interferon activation site) transcription elements through the actions of the STAT1/STAT2 heterodimer. In contrast, type II IFNs can transactivate a gene only if it contains the GAS element by means of the STAT1 homodimer [25,39].

In our DNA microarray dataset, the *IFNB1* (IFN-β) gene is upregulated 80%. It belongs to the type I IFNs which can activate the JAK/STAT1 and STAT2 pathways to regulate the expression of genes encoding chemokines that act downstream in this pathway. We thus suggested that ECPsp may regulate chemokine production by this pathway. Transcriptomic analyses indicate that the upregulation of JAK-STAT1 signaling plays a crucial role in protecting against microbial infection [40,41] by enhancing the expression of genes encoding chemokines (CXCL1, CXCL2, CCL2, and CCL5), a cytokine (IL-6), and components of JAK-STAT1 signaling (IFN regulatory factor 7 [IRF7], IRF9, and STAT1) [42]. Similarly, our investigation showed that genes including IRF7, IFN-induced protein with tetratricopeptides (IFIT1, IFIT2, IFIT3, and IFIT5), myxovirus resistance 1 and 2 (MX1 and MX2), and 2′,5′-oligoadenylate synthetases (OAS1, OAS2, OAS3, and OASL) are upregulated by ECPsp. In all of these instances, transactivation may depend on JAK/STAT signaling. Therefore, we hypothesized that under inflammatory conditions, eosinophils release ECP to damage pathogens and that ECPsp simultaneously induces JAK/STAT signaling to protect host cells from microbial infection and trigger chemokine production that might recruit immune cells, such as macrophages, to inflammatory sites.

## Conclusion

We report a potential new function for ECPsp, which involves the secretion of proinflammatory molecules to promote the migration of macrophages to sites of inflammation. This finding provides clues for researchers to study the roles of ECP and ECPsp under inflammatory conditions. We propose that ECPsp may serve as an effector to induce proinflammatory gene expression and to trigger the recruitment of immune cells, thus eliminating pathogens, removing damaged cells, and repairing bronchial epithelial cells *via* JAK/STAT signaling.

## Methods

### Cells and cell culture

We cultured the human bronchial epithelial cell line BEAS-2B (ATCC CRL-9609) and the mouse macrophage RAW 264.7 cell line in RPMI-1640 (Invitrogen, USA) supplemented with 10% FBS. Human epidermoid carcinoma cell line A431 was cultured in DMEM (GIBCO) supplemented with 10% heat-inactivated fetal bovine serum. All cells were cultured at 37°C in an incubator under 5% CO_2_ and 95% air.

### Transfection

The plasmids pEGFPC1 and pEGFPN1-ECPsp were individually transfected into BEAS-2B cells using TurboFect^TM^ (Thermo Fisher Scientific Inc., USA). For the transfection, 2 × 10^6^ BEAS-2B or A431 cells were seeded onto a 100-mm culture plate. After 24 h in culture (~80-90% confluency), 3 μg of plasmid DNA and 6 μl of TurboFect^TM^ reagent were mixed in 1 ml of serum-free RPMI-1640. The transfection mixture was gently vortexed and incubated for 15 min at room temperature to allow the formation of transfection complexes. The 1-ml transfection mixture was then added drop-wise to the cells and incubated at 37°C for another 48 h, at which point the cells were harvested for RNA isolation and Western blotting.

For plasmid and siRNA co-transfection, 3 × 10^5^ BEAS-2B cells were seeded onto a 60-mm culture plate. After 24 h, the pEGFPN1-ECPsp and siRNA (against STAT1 or STAT2, final concentration: 50 nM) were added into 200 μl jetPRIME® buffer (Polyplus-transfection) and mixed by pipetting. Then, 8 μl jetPRIME® reagent was added. The transfection mixture was gently vortexed, and incubated at room temperature. After 15 min, the transfection mixture was added drop-wise to the cells and incubated at 37°C. The original medium was replaced by fresh one at 24 h post-transfection. After additional 24 h, the cell lysates were harvested for Western blotting.

### RNA extraction and cDNA preparation

Total RNA from BEAS-2B cells was isolated using TRIzol reagent (Invitrogen, USA). Cells that had been cultured in a 100-mm dish were washed with cold PBS. TRIzol reagent (1 ml) was added, and the cells were lysed by pipetting the mixture up and down several times. The cell lysate was then transferred to a 1.5-ml microcentrifuge tube. After the addition of 300 μl of cold chloroform (Sigma, USA), the lysate was gently mixed for 15 seconds, incubated on ice for 15 min, and then centrifuged at 10,000 ×*g* at 4°C for 15 min. The RNA, which remained exclusively in the aqueous phase, was transferred to a new tube. An equal volume of 100% isopropanol was then added and mixed gently by inverting the microcentrifuge tube repeatedly. The precipitated RNA was recovered by centrifugation at 12,000 rpm for 15 min at 4°C. After removal of the supernatant, the RNA pellet was washed twice with 75% ethanol dissolved in diethylpyrocarbonate (DEPC)-treated water and then air-dried. The RNA was dissolved in sterile DEPC-treated water. RT-PCR was used to determine gene expression based on the level of RNA abundance. Total RNA (2 μg) and 1 μl of 10 μM oligo-dT primer (MDBio, Taiwan) were mixed in DEPC-treated water and heated at 70°C for 5 min. The reverse transcription mixture containing 5 μl of Moloney Murine Leukemia Virus (M-MLV) 5× reaction buffer (Promega, USA), 5 μl of 2.5 mM dNTP (2.5 mM each of dATP, dTTP, dCTP, and dGTP; Protech, Taiwan), 1 μl of M-MLV reverse transcriptase (Promega, USA), and DEPC-treated water added to a final volume of 20 μl. The cDNA was generated at 42°C.

### Semi-quantitative RT-PCR

Coding regions of the genes CCL5, CXCL10, CXCL11, CXCL16, STAT1, STAT2, and GAPDH were amplified using the following primer sets: CCL5-F’ (ccctcgctgtcatcctcattg)/CCL5-R’ (gtgacaaagacgactgctgg), CXCL10-F’ (ggcattcaaggagtacctct)/CXCL10-R’ (attcagacatctcttctcac), CXCL11-F’ (agttgttcaaggcttccccatg)/CXCL11-R’ (gggatttaggcatcgttgtcc), CXCL16-F’ (actcagccaggcaatggcaa)/CXCL16-R’ (tccaggaaaggagctggaac), STAT1-F’ (tgcgcgcagaaaagtttcat)/STAT1-R’ (ggattcaaccaaaggagcag), STAT2-F’ (ccagaactggcaggaagctg)/STAT2-R’ (atgtcccggcagaatttccg), and GAPDH-F’ (accacagtccatgccatcac)/GAPDH-R’ (tccaccaccctgttgctgta), respectively. cDNA (1 μl) was used as the template and was mixed with 0.5 μl of each primer (10 μM), 2 μl of dNTP (Protech, Taiwan), 0.25 μl of GoTaq Flexi DNA polymerase (5 U/μl; Promega, USA), 4 μl of 5× Colorless GoTaq reaction buffer (Promega, USA), 1.2 μl of 25 mM MgCl_2_, and sterile deionized water to a final volume of 20 μl. The sample was heated at 95°C for 5 min, followed by 30 cycles, each comprising 95°C for 30 sec, 55°C for 30 sec, and 72°C for 30 sec, and one cycle of 72°C for 10 min in a thermocycler (Multigene, USA).

### DNA microarray analysis

The Human Whole Genome OneArray® v5 (HOA; Phalanx Biotech Group, Taiwan) has in total 30,275 DNA oligonucleotide probes, and each probe has 60 nucleotides in the sense strand. Of these probes, 29,187 probes correspond to the annotated genes in the RefSeq v38 and Ensembl v56 databases. Furthermore, 1,088 control probes are also included for monitoring the sample quality and the hybridization process. Fluorescent aRNA targets were prepared from 1.0 μg total RNA samples using OneArray® Amino Allyl aRNA Amplification Kit (Phalanx Biotech Group, Taiwan) and Cy5 dyes (Amersham Pharmacia, Piscataway, NJ, USA). Fluorescent targets were hybridized to the Human Whole Genome OneArray® with Phalanx hybridization buffer using Phalanx Hybridization System. After 16-hr hybridization at 50°C, non-specific binding targets were washed away by three different washing steps (Wash I: at 42°C for 5 min; Wash II: at 42°C for 5 min and subsequent one at 25°C for 5 min; and Wash III: rinsing the chips for 20 times), and the slides were dried by centrifugation and scanned by an Axon 4000B scanner (Molecular Devices, Sunnyvale, CA, USA). The Cy5 fluorescent intensities of each spot were analyzed by GenePix 4.1 software (Molecular Devices). The signal intensity of each spot was loaded into the Rosetta Resolver System® (Rosetta Biosoftware) for data analysis [43]. The error model of the Rosetta Resolver System® removed both systematic and random errors from the data. We filtered out spots for which the flag was less than zero. Spots that passed the criterion for further consideration were normalized using the 50% media scaling normalization method. The technical repeat data were tested by calculating the Pearson correlation coefficient to assess reproducibility (*r* >0.975). To identify the differentially expressed genes, we calculate the log_2_ ratio of the normalized intensity of treatment sample over intensity of control sample as follows,

(1) $$\text{R}={\text{log}}_{2}\left({\text{I}}_{\text{t}}/{\text{I}}_{\text{c}}\right)$$

where It is one probe intensity of the treatment sample, Ic is one probe intensity of control sample. If a probe P’s R value is bigger than the threshold (usually set as 1), P is regarded as different expression on treatment and control. Furthermore we can calculate the p-value *p* (differential expression) to test the statistical significance of R, which means the probability of null hypothesis that treatment sample and control sample are not differential expression for one probe. The detailed procedure to estimate *p* is formally described in [43]. In our implementation, the spots with *p* <0.05 and R ≥1 were identified as differentially expressed genes for further pathway analysis. The DNA microarray data has been submitted to and approved by the Gene Expression Omnibus Database (GEO) with an accession number of GSE39122.

### String

According to the gene expression profiles derived from DNA microarray, 93 genes upregulated more than 2-fold (log_2_ ≥1) and with the statistic *p* less than 0.05 were selected and assembled as a gene expression subdataset (Table 1). The functional linkage network was generated using STRING 9.0 database [44]. The gene symbols listed in the gene expression subdataset were input into the frame of “multiple names” in STRING and the organism was set *Homo sapiens*. After executing the search, STRING would show the possible genes we input and we had to select correct ones. Then, the functional linkage could be generated using the default setting. Within this completely functional network, the evident area of dense interconnections could be identified. In this network, 93 nodes are connected by 676 edges, and in the dense area, there are 618 edges to connect at least one of the 40 nodes. Although only 43.1% (40/93) of nodes are located in the dense area, there are 91.42% (618/676) edges to connect the nodes in dense area with other nodes.

Furthermore, to analyze the relationship of the functional network, and the TGF-α/EGFR pathway which we discovered previously [22], the genes within the evident area, TGF-α and EGFR were input into STRING again. The setting of STRING database is also default. Then, the functional network linked evident area and TGF-α/EGFR pathway could be generated. The figures presented here are redrawn using Cytoscape software which could calculate the numbers of nodes and edges efficiently.

### NOA

The NOA ( http://app.aporc.org/NOA) database was designed for identifying the enrichment of gene ontology based on biological networks as classified by systems biology [24]. According to microarray data (Table 1), the 93 genes of interest were input into and analyzed by the NOA server to determine the relationship of network ontology.

### Western blotting

After SDS-PAGE analysis, the proteins were transferred onto a polyvinylidene fluoride (PVDF) membrane (Pall, Pensacola). The membrane was incubated in 5% fetal bovine serum (FBS) in PBST (1X PBS and 0.1% Tween 20) at room temperature for 1 h and then the diluted antibody (anti-STAT1 mAb, 1:2,500 or anti-STAT2 mAb, 1:2,500, Santa Cruz, USA) in 5% FBS/PBST was added to react with the target proteins with shaking at 4°C for 16 h. The membrane was washed with PBST three times for 15 min each. The secondary antibody (anti-rabbit IgG conjugated with HRP, 1:5,000, Jackson, USA) was diluted in 3% Milk/PBST with shaking at 25°C. After 1.5 h, the membrane was washed with PBST for three times and the target protein was visualized by addition of the mixture of reagents A and B (Pierce) to the PVDF membrane, and exposed by the ImageQuant^TM^ LAS 4000 (GE Healthcare).

### Macrophage migration assay

Migration assays were performed in Transwell plates (Corning Costar, USA) with a 6.5-mm diameter and an 8-μm pore size for the membrane. Conditioned medium was prepared before adding RAW 264.7 cells into the top well. The BEAS-2B cells were seeded in 60-mm dishes. After 24 h, both plasmids (pEGFP-C1 and pEGFP-N1-ECPsp) were transfected into BEAS-2B cells (as described above) for 6 h. The culture medium containing the transfection mixture was removed, and fresh RPMI-1640 medium supplemented with 10% FBS was added for a further 18-h incubation at 37°C in a humidified 5% CO_2_ incubator. The culture medium was removed, and fresh serum-free RPMI-1640 was added prior to incubation at 37°C for 24 h. Three hundred microliters of the serum-free medium was collected, centrifuged to remove the debris, and then transferred into the bottom wells. At the same time, 1.5 × 10^5^ RAW 264.7 cells were transferred to each top well. After incubation for 8 h at 37°C, cells in the top wells were fixed using 3.7% formaldehyde for 5 min and then stained with 0.05% crystal violet for 30 min, with three further rinses with PBS. The number of cells migrating to the lower membrane surface was counted, and these data were used for further two-tailed student’s *t* test analysis.

## Abbreviations

ECP: Eosinophil cationic protein; EGFR: Epidermal growth factor receptor; IFN: Interferon; NOA: Network Ontology Analysis; RNase: Ribonuclease; SP: Signal peptidase; ECPsp: Signal peptide of ECP; SPP: Signal peptide peptidase; STAT: Signal transducers and activators of transcription; TGF-α: Transforming growth factor-alpha.

## Competing interests

The authors declared that no competing interests exist.

## Authors’ contributions

YSL performed the RNA isolation and the network analyses. PWT and CYL assisted with the transcriptomic analyses. TF assisted with the RT-PCR experiments. YW analyzed the network. CHH assisted with the migration assay. MDTC and TWP were involved deeply in experimental design and discussion. CFH assisted with microarray analysis. HTC lead the project, designed the experiments, and wrote the manuscript. All authors read and approved the final manuscript.

## Supplementary Material

Additional file 1 **Figure S1.** The mRNA and protein levels of STAT1 and STAT2 were upregulated by ECPsp. The protein levels of STAT1 (A) and STAT2 (B), and the mRNA levels of STAT1 (C) and STAT2 (D) were analyzed using Western blotting and semi-quantitative RT-PCR, respectively. (**, *p* < 0.01; * *p*< 0.05). (DOCX 599 kb) Click here for file

## References

[B1] Olsson I Venge P Cationic proteins of human granulocytes. II. Separation of the cationic proteins of the granules of leukemic myeloid cells Blood 1974 44 2 235 246 4211856

[B2] Mallorqui-Fernandez G Pous J Peracaula R Aymami J Maeda T Tada H Yamada H Seno M de Llorens R Gomis-Ruth FX Three-dimensional crystal structure of human eosinophil cationic protein (RNase 3) at 1.75 A resolution J Mol Biol 2000 300 5 1297 1307 10.1006/jmbi.2000.3939 10903870

[B3] Peterson CG Jornvall H Venge P Purification and characterization of eosinophil cationic protein from normal human eosinophils Eur J Haematol 1988 40 5 415 423 3132400 10.1111/j.1600-0609.1988.tb00850.x

[B4] Malabanan AO Turner AK Rosenberg IN Holick MF Oncogenic osteomalacia: clinical presentation, densitometric findings, and response to therapy J Clin Densitom 1998 1 1 77 80 10.1385/JCD:1:1:77 15304916

[B5] Rosenberg HF Recombinant human eosinophil cationic protein. Ribonuclease activity is not essential for cytotoxicity J Biol Chem 1995 270 14 7876 7881 7713881 10.1074/jbc.270.14.7876

[B6] Makarov AA Ilinskaya ON Cytotoxic ribonucleases: molecular weapons and their targets FEBS Lett 2003 540 1–3 15 20 12681476 10.1016/s0014-5793(03)00225-4

[B7] Rosenberg HF RNase A ribonucleases and host defense: an evolving story J Leukoc Biol 2008 83 5 1079 1087 10.1189/jlb.1107725 18211964 PMC2692241

[B8] Cheng GZ Li JY Li F Wang HY Shi GX Human ribonuclease 9, a member of ribonuclease A superfamily, specifically expressed in epididymis, is a novel sperm-binding protein Asian J Androl 2009 11 2 240 251 10.1038/aja.2008.30 19137000 PMC3735023

[B9] Rosenberg HF Ackerman SJ Tenen DG Human eosinophil cationic protein. Molecular cloning of a cytotoxin and helminthotoxin with ribonuclease activity J Exp Med 1989 170 1 163 176 10.1084/jem.170.1.163 2473157 PMC2189377

[B10] Cho S Beintema JJ Zhang J The ribonuclease A superfamily of mammals and birds: identifying new members and tracing evolutionary histories Genomics 2005 85 2 208 220 10.1016/j.ygeno.2004.10.008 15676279

[B11] Carreras E Boix E Navarro S Rosenberg HF Cuchillo CM Nogues MV Surface-exposed amino acids of eosinophil cationic protein play a critical role in the inhibition of mammalian cell proliferation Mol Cell Biochem 2005 272 1–2 1 7 16010966 10.1007/s11010-005-4777-2

[B12] Carreras E Boix E Rosenberg HF Cuchillo CM Nogues MV Both aromatic and cationic residues contribute to the membrane-lytic and bactericidal activity of eosinophil cationic protein Biochemistry 2003 42 22 6636 6644 10.1021/bi0273011 12779318

[B13] Fan TC Chang HT Chen IW Wang HY Chang MD A heparan sulfate-facilitated and raft-dependent macropinocytosis of eosinophil cationic protein Traffic 2007 8 12 1778 1795 10.1111/j.1600-0854.2007.00650.x 17944807

[B14] Gleich GJ Mechanisms of eosinophil-associated inflammation J Allergy Clin Immunol 2000 105 4 651 663 10.1067/mai.2000.105712 10756213

[B15] Winterkamp S Raithel M Hahn EG Secretion and tissue content of eosinophil cationic protein in Crohn’s disease J Clin Gastroenterol 2000 30 2 170 175 10.1097/00004836-200003000-00009 10730922

[B16] Pegorier S Wagner LA Gleich GJ Pretolani M Eosinophil-derived cationic proteins activate the synthesis of remodeling factors by airway epithelial cells J Immunol 2006 177 7 4861 4869 16982928 10.4049/jimmunol.177.7.4861

[B17] Domachowske JB Dyer KD Adams AG Leto TL Rosenberg HF Eosinophil cationic protein/RNase 3 is another RNase A-family ribonuclease with direct antiviral activity Nucleic Acids Res 1998 26 14 3358 3363 10.1093/nar/26.14.3358 9649619 PMC147714

[B18] Lehrer RI Szklarek D Barton A Ganz T Hamann KJ Gleich GJ Antibacterial properties of eosinophil major basic protein and eosinophil cationic protein J Immunol 1989 142 12 4428 4434 2656865

[B19] Molina HA Kierszenbaum F Hamann KJ Gleich GJ Toxic effects produced or mediated by human eosinophil granule components on Trypanosoma cruzi AmJTrop Med Hyg 1988 38 2 327 334 10.4269/ajtmh.1988.38.327 2451444

[B20] Young JD Peterson CG Venge P Cohn ZA Mechanism of membrane damage mediated by human eosinophil cationic protein Nature 1986 321 6070 613 616 10.1038/321613a0 2423882

[B21] Wu CM Chang MD Signal peptide of eosinophil cationic protein is toxic to cells lacking signal peptide peptidase Biochem Biophys Res Commun 2004 322 2 585 592 10.1016/j.bbrc.2004.07.160 15325270

[B22] Chang HT Kao YL Wu CM Fan TC Lai YK Huang KL Chang YS Tsai JJ Chang MD Signal peptide of eosinophil cationic protein upregulates transforming growth factor-alpha expression in human cells J Cell Biochem 2007 100 5 1266 1275 10.1002/jcb.21120 17063486

[B23] Woschnagg C Rubin J Venge P Eosinophil cationic protein (ECP) is processed during secretion J Immunol 2009 183 6 3949 3954 10.4049/jimmunol.0900509 19692640

[B24] Wang J Huang Q Liu ZP Wang Y Wu LY Chen L Zhang XS NOA: a novel Network Ontology Analysis method Nucleic Acids Res 2011 39 13 e87 10.1093/nar/gkr251 21543451 PMC3141273

[B25] Schindler C Plumlee C Inteferons pen the JAK-STAT pathway Semin Cell Dev Biol 2008 19 4 311 318 10.1016/j.semcdb.2008.08.010 18765289 PMC2741134

[B26] Canaff L Zhou X Hendy GN The proinflammatory cytokine, interleukin-6, up-regulates calcium-sensing receptor gene transcription via Stat1/3 and Sp1/3 J Biol Chem 2008 283 20 13586 13600 10.1074/jbc.M708087200 18348986

[B27] Qin S Rottman JB Myers P Kassam N Weinblatt M Loetscher M Koch AE Moser B Mackay CR The chemokine receptors CXCR3 and CCR5 mark subsets of T cells associated with certain inflammatory reactions J Clin Invest 1998 101 4 746 754 10.1172/JCI1422 9466968 PMC508621

[B28] Lukacs NW Role of chemokines in the pathogenesis of asthma Nat Rev Immunol 2001 1 2 108 116 10.1038/35100503 11905818

[B29] Matsumura S Wang B Kawashima N Braunstein S Badura M Cameron TO Babb JS Schneider RJ Formenti SC Dustin ML Radiation-induced CXCL16 release by breast cancer cells attracts effector T cells J Immunol 2008 181 5 3099 3107 18713980 10.4049/jimmunol.181.5.3099 PMC2587101

[B30] Powell N Humbert M Durham SR Assoufi B Kay AB Corrigan CJ Increased expression of mRNA encoding RANTES and MCP-3 in the bronchial mucosa in atopic asthma Eur Respir J 1996 9 12 2454 2460 10.1183/09031936.96.09122454 8980953

[B31] Kapp A Zeck-Kapp G Czech W Schopf E The chemokine RANTES is more than a chemoattractant: characterization of its effect on human eosinophil oxidative metabolism and morphology in comparison with IL-5 and GM-CSF J Invest Dermatol 1994 102 6 906 914 10.1111/1523-1747.ep12383399 7516398

[B32] Meurer R Van Riper G Feeney W Cunningham P Hora D Jr Springer MS MacIntyre DE Rosen H Formation of eosinophilic and monocytic intradermal inflammatory sites in the dog by injection of human RANTES but not human monocyte chemoattractant protein 1, human macrophage inflammatory protein 1 alpha, or human interleukin 8 J Exp Med 1993 178 6 1913 1921 10.1084/jem.178.6.1913 7504053 PMC2191290

[B33] de Nadai P Chenivesse C Gilet J Porte H Vorng H Chang Y Walls AF Wallaert B Tonnel AB Tsicopoulos A CCR5 usage by CCL5 induces a selective leukocyte recruitment in human skin xenografts in vivo J Invest Dermatol 2006 126 9 2057 2064 10.1038/sj.jid.5700369 16778803

[B34] Cui Q Ma Y Jaramillo M Bari H Awan A Yang S Zhang S Liu L Lu M O’Connor-McCourt M A map of human cancer signaling Mol Syst Biol 2007 3 152 18091723 10.1038/msb4100200 PMC2174632

[B35] Caporale M Arnaud F Mura M Golder M Murgia C Palmarini M The signal peptide of a simple retrovirus envelope functions as a posttranscriptional regulator of viral gene expression J Virol 2009 83 9 4591 4604 10.1128/JVI.01833-08 19244321 PMC2668452

[B36] Martoglio B Graf R Dobberstein B Signal peptide fragments of preprolactin and HIV-1 p-gp160 interact with calmodulin EMBO J 1997 16 22 6636 6645 10.1093/emboj/16.22.6636 9362478 PMC1170268

[B37] Medoff BD Sauty A Tager AM Maclean JA Smith RN Mathew A Dufour JH Luster AD IFN-gamma-inducible protein 10 (CXCL10) contributes to airway hyperreactivity and airway inflammation in a mouse model of asthma J Immunol 2002 168 10 5278 5286 11994485 10.4049/jimmunol.168.10.5278

[B38] de Weerd NA Samarajiwa SA Hertzog PJ Type I interferon receptors: biochemistry and biological functions J Biol Chem 2007 282 28 20053 20057 10.1074/jbc.R700006200 17502368

[B39] Platanias LC Mechanisms of type-I- and type-II-interferon-mediated signalling Nat Rev Immunol 2005 5 5 375 386 10.1038/nri1604 15864272

[B40] Dupuis S Dargemont C Fieschi C Thomassin N Rosenzweig S Harris J Holland SM Schreiber RD Casanova JL Impairment of mycobacterial but not viral immunity by a germline human STAT1 mutation Science 2001 293 5528 300 303 10.1126/science.1061154 11452125

[B41] Dupuis S Jouanguy E Al-Hajjar S Fieschi C Al-Mohsen IZ Al-Jumaah S Yang K Chapgier A Eidenschenk C Eid P Impaired response to interferon-alpha/beta and lethal viral disease in human STAT1 deficiency Nat Genet 2003 33 3 388 391 10.1038/ng1097 12590259

[B42] Lad SP Fukuda EY Li J de la Maza LM Li E Up-regulation of the JAK/STAT1 signal pathway during Chlamydia trachomatis infection J Immunol 2005 174 11 7186 7193 15905563 10.4049/jimmunol.174.11.7186

[B43] Weng L Dai H Zhan Y He Y Stepaniants SB Bassett DE Rosetta error model for gene expression analysis Bioinformatics 2006 22 9 1111 1121 10.1093/bioinformatics/btl045 16522673

[B44] von Mering C Jensen LJ Kuhn M Chaffron S Doerks T Kruger B Snel B Bork P STRING 7--recent developments in the integration and prediction of protein interactions Nucleic Acids Res 2007 35 Database issue D358 D362 17098935 10.1093/nar/gkl825 PMC1669762

