# Supplementary material for: Chemoattraction of macrophages by secretory molecules derived from cells expressing the signal peptide of eosinophil cationic protein
Source: BMC Syst Biol. 2012 Aug 20;6:105. doi: 10.1186/1752-0509-6-105 (PMC3478170; doi:10.1186/1752-0509-6-105)
Supplement: Additional file 1 — Figure S1. The mRNA and protein levels of STAT1 and STAT2 were upregulated by ECPsp. The protein levels of STAT1 (A) and STAT2 (B), and the mRNA levels of STAT1 (C) and STAT2 (D) were analyzed using Western blotting and semi-quantitative RT-PCR, respectively. (**, p < 0.01; * p< 0.05). (DOCX 599 kb) [file 1752-0509-6-105-S1.docx]

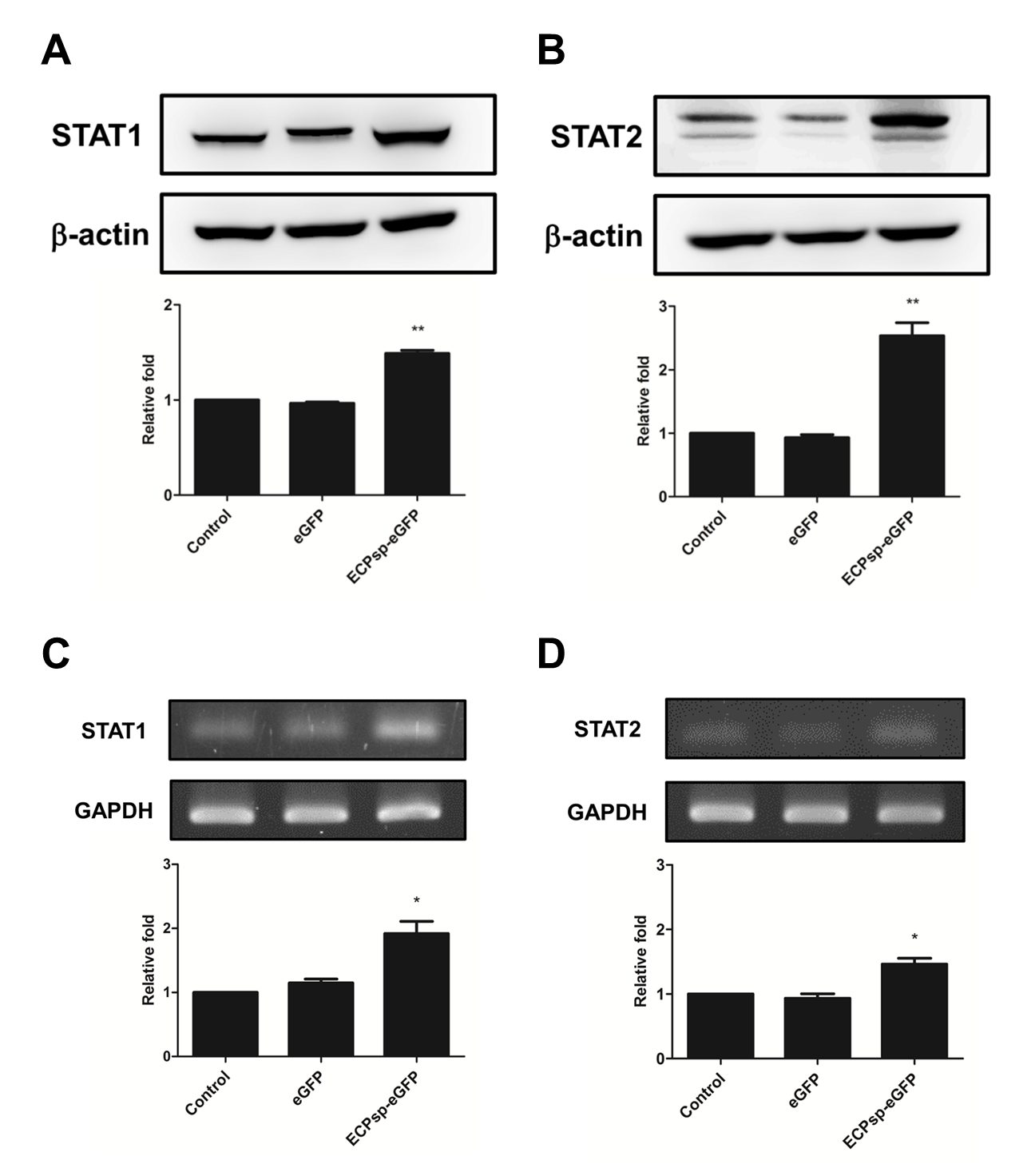


Supplementary Figure 1. The mRNA and protein levels of STAT1 and STAT2 were upregulated by ECPsp. The protein levels of STAT1 (A) and STAT2 (B), and the mRNA levels of STAT1 (C) and STAT2 (D) were analyzed using Western blotting and semi-quantitative RT-PCR, respectively. (**, *p* < 0.01; * *p*< 0.05)
